# Comparison of MIB‐1‐Specific Membrane Staining in Hyalinising Trabecular Tumor Using Mainstream Automated Immunohistochemical Staining Platforms

**DOI:** 10.1002/jcla.25113

**Published:** 2024-10-24

**Authors:** Bo Hong, Yanfei Xu, Yufei Xiao, Xiaoyan Yu

**Affiliations:** ^1^ Department of Pathology The Second Affiliated Hospital, Zhejiang University School of Medicine Hangzhou Zhejiang People's Republic of China; ^2^ Department of Pathology Quzhou Second People's Hospital Quzhou Zhejiang People's Republic of China; ^3^ Department of Clinical Laboratory The Second Affiliated Hospital, Zhejiang University School of Medicine Hangzhou Zhejiang People's Republic of China

**Keywords:** diagnosis, hyalinising trabecular tumor, immunohistochemistry, Ki‐67, MIB‐1

## Abstract

**Background:**

MIB‐1, a monoclonal antibody against Ki‐67, exhibits specific membrane staining in the immunohistochemistry of hyalinising trabecular tumor (HTT). This specific staining pattern is crucial in diagnosing HTT. Although manual immunohistochemical staining remains the established method for MIB‐1 staining, this process is complicated, inconsistent, and prone to false negatives.

**Methods:**

This study aimed to explore whether the classical reaction pattern can be replicated by utilizing the current mainstream automated immunohistochemical staining platforms. Furthermore, we examined the effect of different conditions on staining efficiency and their value in clinical diagnosis assistance.

**Results:**

Specimens obtained from eight and six cases of HTT and non‐HTT, respectively, from a single center were stained using the manual staining method and the Dako Autostainer Link 48 (AS48), Dako Omnis, Ventana BenchMark ULTRA, and Leica BOND‐III automated immunohistochemical staining platforms. The Autostainer Link 48 was found to be the most stable staining platform, while the BenchMark ULTRA with primary antibody incubation at room temperature (RT) and the Omnis platform with antigen retrieval at pH 9.0 were able to reproduce membrane‐positive staining for MIB‐1 in the HTT specimens.

**Conclusions:**

Our results offer crucial reference value for clinical diagnostic assistance.

## Graphical abstract

The Autostainer Link 48 was found to be the most stable staining platform for MIB‐1 in HTT. The BenchMark ULTRA with primary antibody incubation at room temperature (RT) and the Omnis platform with antigen retrieval at pH 9.0 were able to reproduce membrane‐positive staining for MIB‐1 in the HTT specimens.

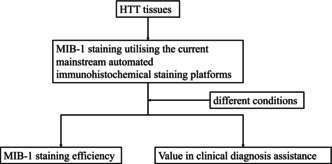

## 1 Introduction

Hyalinising trabecular tumor (HTT) was first described by Carney et al. in 1987 as a rare follicular thyroid tumor [1]. Although HTT is generally considered benign, its morphological diagnosis can be challenging [2] owing to its similar appearance to papillary thyroid carcinoma (PTC) [3, 4]. Additionally, HTT can be easily confused with medullary thyroid carcinoma (MTC) due to the presence of amyloid transparent material in the tumor [5]. In terms of immunohistochemistry (IHC), these tumor cells show a characteristic cell membrane‐positive reaction for MIB‐1 [6]. MIB‐1 is a monoclonal antibody against Ki‐67, a cell proliferation marker prevalent across all cell cycle phases [7]. MIB‐1 was first identified by Hirokawa et al. as a promising marker for establishing HTT diagnosis [8]. The 2022 WHO classification further emphasized the importance of Ki‐67 membrane staining as a supportive criterion for HTT diagnosis [9], though its absence does not exclude the diagnosis. This feature provides additional clinical evidence but should not be used as a sole diagnostic criterion.

The conventional manual staining method is considered the standard method for MIB‐1 immunohistochemical staining and must be performed at room temperature [10]. Currently, numerous institutions are utilizing automated immunohistochemical platforms, which simplify the staining process and provide more consistent and reproducible results. However, previous study findings have suggested that automated immunohistochemical staining systems do not provide accurate MIB‐1 membrane staining results for detecting HTT [10, 11, 12]. Therefore, this study aimed to determine the optimal conditions and methods for achieving membrane‐positive staining for MIB‐1 expression in HTT cases using various automated immunohistochemical staining platforms.

## 2 Materials and Methods

### 2.1 Tissue Specimens

This study included eight thyroid specimens that were surgically resected and histologically diagnosed as HTT at the Second Affiliated Hospital of Zhejiang University School of Medicine between 2022 and 2023. The patients had a median age of 45 years (range, 24–65 years), comprising one male and six females. The tumor sizes of the patients ranged from 0.2 to 3 cm. Additionally, we included six non‐HTT cases, consisting of four PTCs and one case each of noninvasive follicular thyroid neoplasm with papillary‐like nuclear features (NIFTP) and MTC.

### 2.2 Immunohistochemistry

The Ki‐67 used for IHC was supplied by Dako (Glostrup, Denmark; item code: M7240, clone number: MIB‐1), with all specimens undergoing staining at a 1:100 dilution ratio.

The automated staining platforms that were employed included the BOND‐III (Leica Biosystems, Wetzlar, Germany), BenchMark ULTRA (Ventana, Roche Diagnostics, Basel, Switzerland), Omnis (Dako, Agilent Pathology Solutions, CA, US), and Autostainer Link 48 (Dako, Agilent Pathology Solutions, CA, US) systems. In particular, the Autostainer Link 48 is a semi‐automated staining system in which antigen retrieval and staining reactions are performed separately. Additionally, routine processing, neutral buffered formalin fixation, paraffin embedding, tissue sectioning at 3‐μm thickness, oven baking at 70°C for 30 min, subsequent antigen retrieval, antigen detection, and hematoxylin counterstaining were all performed on the respective equipment.

For antigen retrieval, Dako Autostainer Link 48, Leica Bond III, and Dako Omnis used solutions with pH 6.0 and pH 9.0. Dako Autostainer Link 48 and Leica Bond III allowed primary antibody incubation only at room temperature, while Dako Omnis was set to 32°C. Ventana BenchMark ULTRA primarily uses an alkaline Cell Conditioning Solution (CC1) for heat‐induced epitope retrieval, and in our experiments, we also tested an acidic Cell Conditioning Solution (CC2). However, the results for DAB staining and hematoxylin counterstaining were suboptimal with CC2, so these results are excluded from further analysis. Notably, Ventana BenchMark ULTRA permits adjustable temperatures for primary antibody incubation, which we tested at room temperature and 37°C (Table 1). Manual staining was conducted using a citrate retrieval solution at pH 6.0, with antibody incubation at room temperature.

**TABLE 1:** Immunohistochemical staining protocols to detect cell membranous immunoreactivity for MIB‐1 in the HTT specimens.

Retrieval solution	BOND III		BenchMark ULTRA		Omnis		Autostainer Link 48	
	ER1 (pH 6.0)	ER2 (pH 9.0)	CC1 (pH 8.5)	CC1 (pH 8.5)	TRS pH 9.0	TRS pH 6.0	TRS pH 9.0	TRS pH 6.0
Retrieval temperature	100°C	97°C	100°C	100°C	97°C	97°C	97°C	97°C
Retrieval time	HIER 30'	HIER 20'	HIER 64'	HIER 64'	HIER 30'	HIER 30'	HIER 20'	HIER 20'
Incubation temperature	RT	RT	RT	37°C	32°C	32°C	RT	RT
Incubation time	15'	15'	32'	32'	20'	20'	20'	20'
Detection system	Bond Polymer Refine Detection	Bond Polymer Refine Detection	UltraView Universal DAB Detection Kit	UltraView Universal DAB Detection Kit	EnVision Flex+LINKER	EnVision Flex+LINKER	EnVision Flex+LINKER	EnVision Flex+LINKER

Abbreviations: ER1, Leica BOND Epitope Retrieval Solution 1; ER2, Leica BOND Epitope Retrieval Solution 2; HIER, heat‐induced epitope retrieval; RT, room temperature (25°C); TRS, Dako Target Retrieval Solution; ULTRA CC1, Ventana ULTRA Cell Conditioning Solution.

### 2.3 Quantitative Analysis of Manual IHC Staining

Two pathologists, blinded to the clinical features, conducted a quantitative assessment of the manual IHC staining results. The assessed parameters included the following:

Staining intensity: This was evaluated on a scale from 0 to 3, where 0 represented no staining, 1 indicated weak staining, 2 moderate staining, and 3 strong staining.

Background staining and non‐specific staining: These were assessed to ensure the specificity of the staining reaction. No nuclear staining should be present, while cytoplasmic and membranous staining are acceptable.

Overall quality of the staining reaction: This factor considered the uniformity and consistency of staining across the tissue sections, taking into account both the intensity and localization of the staining.

For the classification of positive tumor staining, tumors were considered positive if they displayed complete or incomplete moderate membrane staining observed in more than 30% of tumor cells. This was readily appreciated using a low‐power objective, providing a robust criterion for determining positive expression across different automated staining platforms.

## 3 Results

### 3.1 Immunohistochemical Results

**FIGURE 1:**
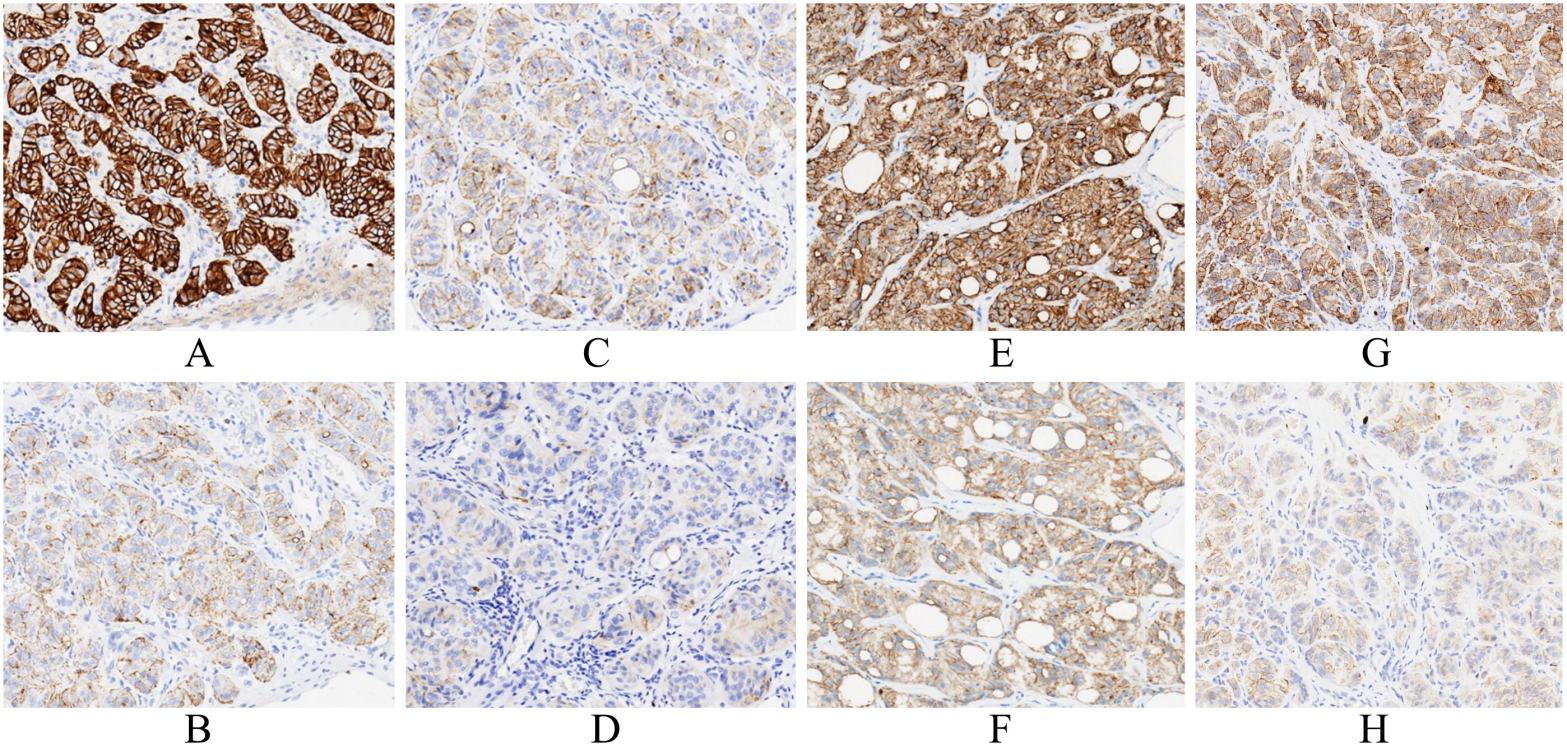
Immunostaining of MIB‐1 in the hyalinising trabecular tumor specimens using four automated platforms under various conditions. (A) AS48 with Target Retrieval Solution (TRS) at pH 9; (B) AS48 with TRS at pH 6; (C) BOND‐III with Epitope Retrieval Solution (ER) 1 at pH 6.0; (D) BOND‐III with ER2 at pH 9.0; (E) BenchMark ULTRA with incubation temperature at room temperature (RT); (F) BenchMark ULTRA with incubation temperature at 37°C; (G) Omnis with TRS at pH 9; and (H) Omnis with TRS at pH 6.

In addition to the manual immunohistochemical staining method, all tests were performed on the previously mentioned automated immunohistochemical staining platforms with varying conditions. In the BOND‐III platform, antibodies were incubated at room temperature, exhibiting weak or essentially no membrane staining using epitope retrieval solution (ER2, pH 9.0) and weak‐to‐moderate membrane staining using ER1 (pH 6.0). IHC on the Ventana BenchMark ULTRA platform was performed using CC1 (pH 8.5), while the primary antibodies were incubated at room temperature and 37°C. The staining intensity was found to be significantly stronger at room temperature than at 37°C. In the Dako Omnis platform, the antibody incubation temperature was fixed at 32°C. The results showed weak to essentially no membrane staining using the target retrieval solution (TRS) at pH 6.0 and weak‐to‐moderate positive staining via the TRS at pH 9.0. Lastly, the Dako Autostainer Link 48 platform is a semi‐automated staining system with procedures most closely resembling the manual staining method. In this process, all assay steps were performed at room temperature without any chemical or physical ‘overlay’, and antigen retrieval was conducted in a water bath using the PT‐Link pre‐treatment method. The staining intensity in the Autostainer Link 48 platform was generally stronger with the TRS at pH 9.0 than at pH 6.0 (Figure 1).

### 3.2 MIB‐1 Membrane Expression Results

The various automated staining platforms were scored according to the conditions that achieved optimal membrane‐positive reactions for MIB‐1. The comparison revealed that the Dako Autostainer Link 48 and the Ventana BenchMark ULTRA with primary antibody incubation at room temperature and the Dako Omnis with antigen retrieval at pH 9.0 were able to reproduce the membrane‐staining reaction for MIB‐1 in the HTT specimens. However, the Dako Omnis with antigen retrieval at pH 6.0 and the Leica BOND‐III could not replicate or only partially replicated the membrane‐staining reaction. All these findings have important reference value for clinical diagnosis assistance.

Additionally, our examination of the membrane‐staining reaction for MIB‐1 using the automated immunohistochemical staining platforms revealed that the antigen retrieval method and incubation temperature as well as the detection system and detection platform are crucial factors affecting the membrane‐positive expression of MIB‐1 in HTT.

### 3.3 Comparison of the Diagnostic Value of Various Automated Staining Platforms Under Different Conditions

**FIGURE 2:**
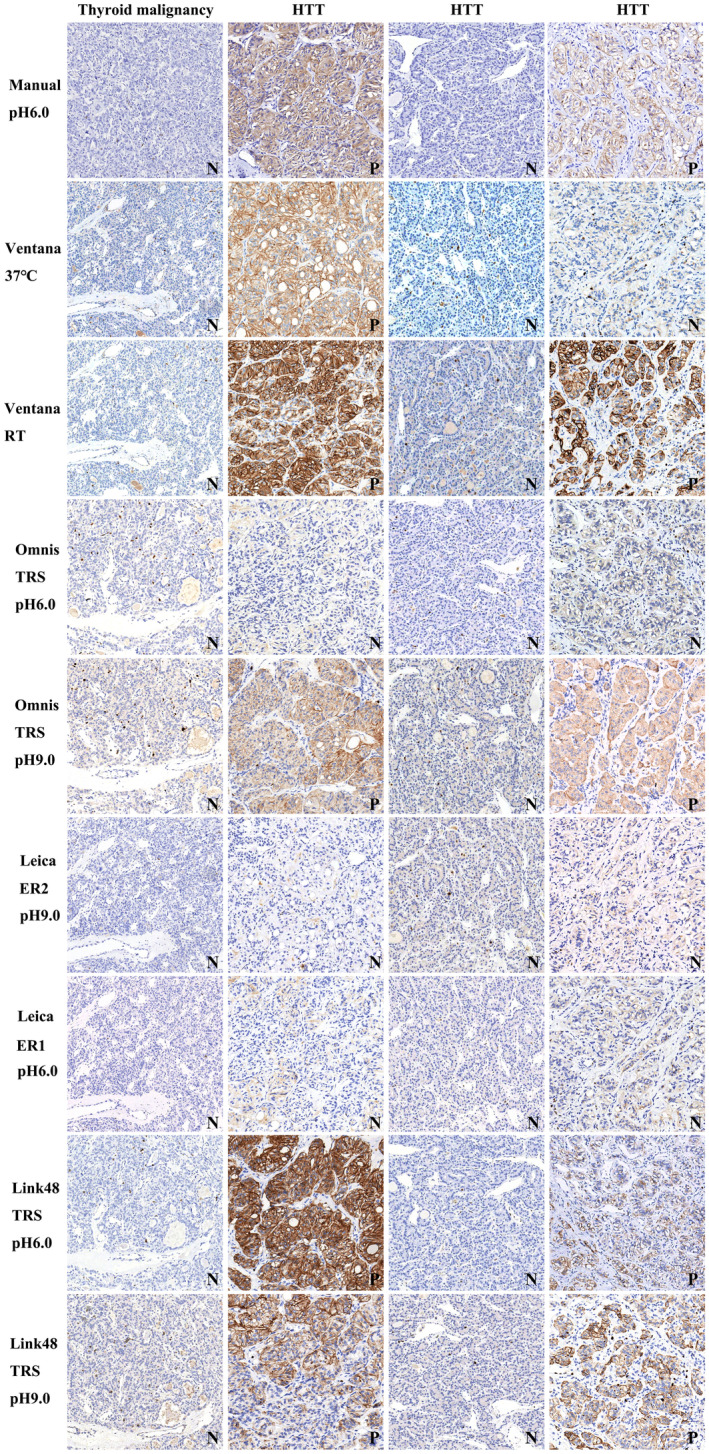
Comparison of the staining results obtained using the various immunohistochemical staining platforms. Each column represents staining of the same tissue sample across different platforms. “P” denotes positive staining, “N” indicates negative staining. The figure illustrates the variability in staining patterns and intensities across the platforms, allowing for a side‐by‐side comparison of their performance.

**FIGURE 3:**
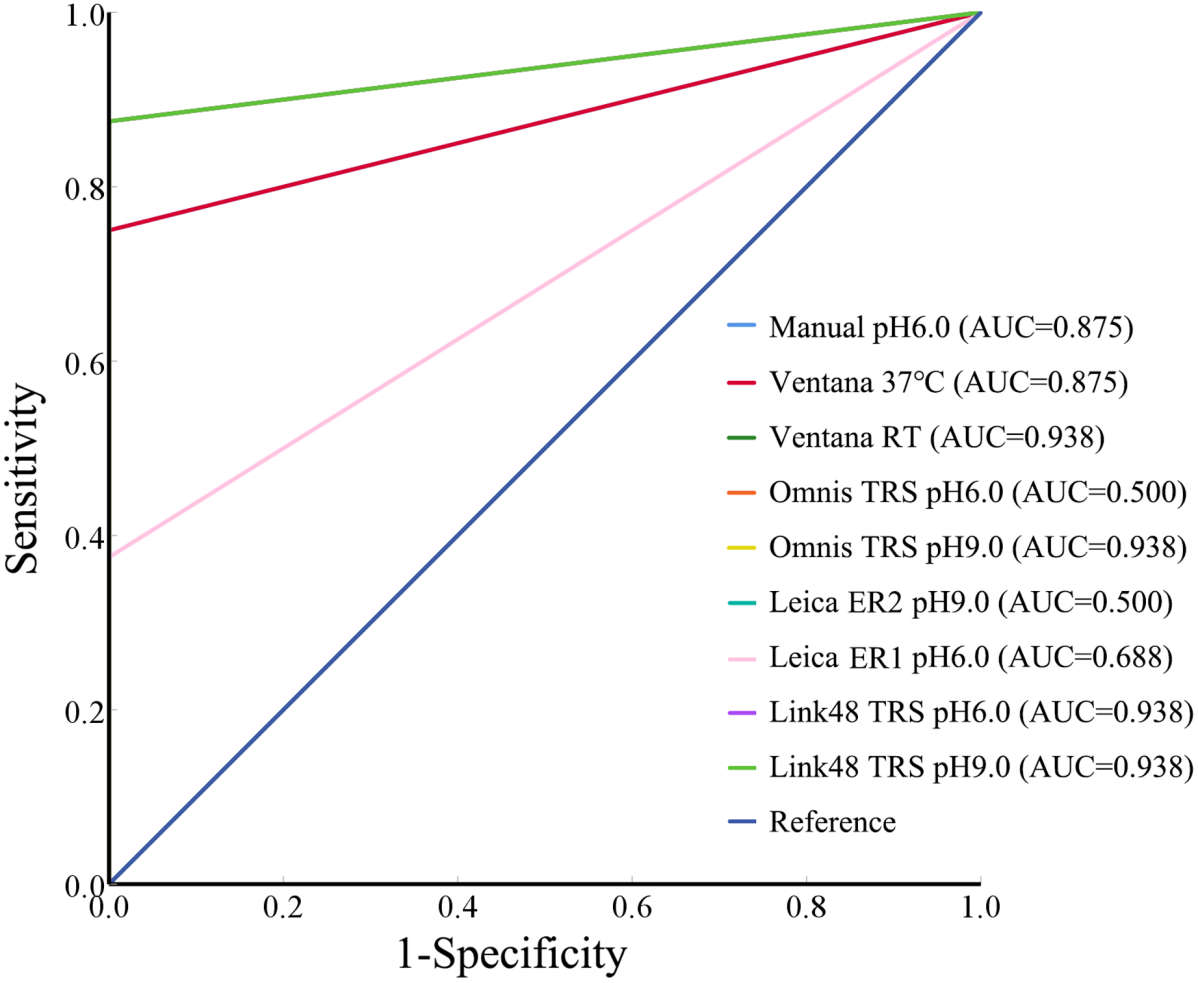
Receiver Operating Characteristic (ROC) curves of the various immunohistochemical staining platforms.

None of the automated staining methods produced false positives, whereas certain instances of false negatives were detected (Figure 2). The receiver operating characteristic (ROC) curve and area under the curve (AUC) of the various automated staining platforms are shown in Figure 3. As demonstrated in Table 2, the conditions of Ventana (RT), Omnis (TRS, pH 9.0), Link 48 (TRS, pH 6.0), and Link 48 (TRS, pH 9.0) resulted in the best staining results, with the Dako Autostainer Link 48 identified as the most stable staining platform.

**TABLE 2:** ROC curves of the manual staining method and various automated immunohistochemical staining platforms.

*p*	Manual (pH 6.0)	Ventana (37°C)	Ventana (RT)	Omnis (TRS, pH 6.0)	Omnis (TRS, pH 9.0)	Leica (ER2, pH 9.0)	Leica (ER1, pH 6.0)	Link 48 (TRS, pH 6.0)	Link 48 (TRS, pH 9.0)
Manual (pH 6.0)		1.000	0.317	0.000	0.317	0.000	0.040	0.317	0.317
Ventana (37°C)	1.000		0.317	0.000	0.317	0.000	0.040	0.317	0.317
Ventana (RT)				0.000	1.000	0.000	0.008	1.000	1.000
Omnis (TRS, pH 6.0)					0.000	1.000	0.040	0.000	0.000
Omnis (TRS, pH 9.0)						0.000	0.008	1.000	1.000
Leica (ER2, pH 9.0)							0.040	0.000	0.000
Leica (ER1, pH 6.0)								0.008	0.008
Link 48 (TRS, pH 6.0)									1.000
Link 48 (TRS, pH 9.0)									

Abbreviations: ER1, Leica BOND Epitope Retrieval Solution 1; ER2, Leica BOND Epitope Retrieval Solution 2; RT, room temperature (25°C); TRS, Dako Target Retrieval Solution.

## 4 Discussion

The specific tumor membrane‐positive reaction for MIB‐1 in HTT was first identified by Hirokawa et al. in 1995, with no other thyroid tumors displaying this staining pattern [6, 8, 13, 14, 15]. Previous studies have suggested that the membrane‐positive expression of MIB‐1 could be a cross‐reaction of epitope expression on the cell membrane or an artefactual phenomenon [10, 16]. Although the membrane‐positive expression of MIB‐1 has also been reported in breast cancer [17, 18], salivary gland pleomorphic adenoma [19], sclerosing haemangioma of the lung [20, 21], renal oncocytoma, and sarcomatoid pleural mesothelioma [10], these patterns were not clinically significant or reproducible.

Incorporating the latest WHO classification, it is clear that Ki‐67 membrane staining serves as an important but supplementary feature in the diagnosis of HTT [9]. The absence of this staining does not exclude HTT, and it should not be the sole diagnostic criterion. Clinically, the presence of membranous Ki‐67 staining can assist in confirming HTT when combined with other histopathological features [8].

Considering that automated immunohistochemical staining platforms not only save the required workforce but also follow a standardized staining process [22], an increasing number of laboratories have stopped utilizing manual staining methods. Prior researchers have identified incubation temperature and antigen retrieval conditions as the primary factors affecting staining, suggesting that the membrane staining of MIB‐1 in HTT can only be achieved at room temperature [10, 12, 23]. In contrast, our study results showed that membrane‐positive staining for MIB‐1 could also be attained at 32°C (Dako Omnis platform) and 37°C (Ventana BenchMark ULTRA platform) but with a generally weaker intensity than that at room temperature. Moreover, we found that the staining intensity with a retrieval solution at high pH was usually stronger than that with a retrieval solution at low pH. The Leica BOND‐III was an exception to this phenomenon, exhibiting stronger staining in low pH than in high pH solutions, along with typically weaker staining at room temperature incubation. Therefore, the staining outcome of MIB‐1 appears to be more dependent on the specific staining platform.

## 5 Conclusion

In this study, we revealed that cell membrane‐positive reactions for MIB‐1 in HTT could be achieved using automated immunohistochemical staining platforms, with the Dako Autostainer Link 48 demonstrated as the most stable staining platform. In terms of staining conditions, the Ventana BenchMark ULTRA with primary antibody incubation at room temperature and the Dako Omnis with antigen retrieval at pH 9.0 were able to reproduce membrane‐positive staining for MIB‐1 in HTT. These results offer crucial reference value for clinical diagnostic assistance. However, we recommend that laboratory technicians first conduct pre‐tests to determine the optimal protocols and pathologists should not rely solely on the cell membrane‐positive reaction for MIB‐1 in the diagnosis of HTT.

## Author Contributions

Bo Hong drafted the manuscript and performed the data analysis and interpretation. Xiaoyan Yu collected the tumor tissue specimens and conducted the IHC staining experiments. Yanfei Xu undertook the verification of all pathology and immunohistochemistry staining results. Bo Hong designed the study, provided administrative support and supervision, reviewed the manuscript, and confirmed the authenticity of all raw data. All authors contributed to the study and approved the submitted version of the manuscript.

## Ethics Statement

This study received ethical approval from the Clinical Research Ethics Committee of the Second Affiliated Hospital, Zhejiang University School of Medicine (approval no. 2024‐0455; Hangzhou, China). The requirement for informed consent was waived by the Clinical Research Ethics Committee of the Second Affiliated Hospital, Zhejiang University School of Medicine. All methods were performed in accordance with the relevant guidelines and regulations.

## Consent

The authors have nothing to report.

## Conflicts of Interest

The authors declare no conflicts of interest.

## Data Availability

The datasets generated and analyzed during the current study are available from the corresponding author upon reasonable request.
